# Effects of Chilling on the Structure, Function and Development of Chloroplasts

**DOI:** 10.3389/fpls.2018.01715

**Published:** 2018-11-22

**Authors:** Xiaomin Liu, Yunlin Zhou, Jianwei Xiao, Fei Bao

**Affiliations:** ^1^College of Biological Sciences and Biotechnology, Beijing Forestry University, Beijing, China; ^2^Beijing Key Laboratory of Ornamental Plants Germplasm Innovation & Molecular Breeding, National Engineering Research Center for Floriculture, Beijing Laboratory of Urban and Rural Ecological Environment, Engineering Research Center of Landscape Environment of Ministry of Education, Key Laboratory of Genetics and Breeding in Forest Trees and Ornamental Plants of Ministry of Education, School of Landscape Architecture, Beijing Forestry University, Beijing, China

**Keywords:** chilling, chloroplasts, development, chloroplast structure, ROS, photosynthesis

## Abstract

Chloroplasts are the organelles that perform energy transformation in plants. The normal physiological functions of chloroplasts are essential for plant growth and development. Chilling is a common environmental stress in nature that can directly affect the physiological functions of chloroplasts. First, chilling can change the lipid membrane state and enzyme activities in chloroplasts. Then, the efficiency of photosynthesis declines, and excess reactive oxygen species (ROS) are produced. On one hand, excess ROS can damage the chloroplast lipid membrane; on the other hand, ROS also represent a stress signal that can alter gene expression in both the chloroplast and nucleus to help regenerate damaged proteins, regulate lipid homeostasis, and promote plant adaptation to low temperatures. Furthermore, plants assume abnormal morphology, including chlorosis and growth retardation, with some even exhibiting severe necrosis under chilling stress. Here, we review the response of chloroplasts to low temperatures and focus on photosynthesis, redox regulation, lipid homeostasis, and chloroplast development to elucidate the processes involved in plant responses and adaptation to chilling stress.

## Introduction

Chloroplasts develop from proplastids under different leaf development contexts in monocots and dicots. Chloroplasts have a double membrane envelope that encloses the stroma and thylakoids. As plant organelles, chloroplasts are sensitive to changes in environmental conditions, and they can react quickly – within a few minutes – by movement. Chloroplasts are the exclusive sites of photosynthesis and capture light energy from the sun to produce chemical energy. Moreover, chloroplasts are involved in many other metabolic pathways, including the synthesis of lipids, pigments, plant hormones, and numerous other metabolites. Plants need to maintain a steady-state balance between energy generation and consumption (Suzuki et al., 2012). Once this balance is destroyed, oxidative stress can be induced in cells by promoting the generation and accumulation of reactive oxygen species (ROS), such as singlet oxygen (^1^O_2_), superoxide anion radicals (O_2_^−^), hydrogen peroxide (H_2_O_2_), and hydroxyl radicals (Takahashi and Asada, 1988; Mittler, 2002; Takahashi and Murata, 2008; Miller et al., 2010). For example, limiting CO_2_ fixation by drought, salt or temperature stress can enhance the production of ROS (Mittler et al., 2004). Chloroplasts are a major sites for the production of ROS (Mignolet-Spruyt et al., 2016). ROS cause the oxidation of cellular components, interfere with metabolic activities and affect chloroplast integrity. Plants have developed many strategies to address these phenomena and maintain suitable photosynthetic efficiency. These processes involve a complex chain of redox reactions and the reactive oxygen scavenging system.

Low temperatures, also called chilling, refer to low, but not freezing, temperatures (0–15°C) (Theocharis et al., 2012; Wang S. et al., 2016). Low temperatures can affect plant growth during each stage of life from germination to maturity and limit the distribution of plants throughout the world. Different plants have different capacities to tolerate chilling stress. Many tropical and subtropical plants, including tobacco, rice and maize, cannot survive at chilling temperatures, whereas *Arabidopsis* and some overwintering cereals can continue to grow (Stitt and Hurry, 2002; Wang S. et al., 2016). Early studies reported that a series of physiological and cellular changes occur in plants under chilling conditions, including alterations in the membrane structure, photosynthesis, calcium signals, and metabolism (Lyons, 1973; Theocharis et al., 2012). These changes facilitate plant adaptation to chilling.

Chloroplasts, which are the sites of photosynthesis, can sense low temperatures and are the first and most severely affected organelles in plants (Lee et al., 2007). Chilling affects chloroplast function by inhibiting photosynthesis, inducing changes in the organelle’s ultrastructure, and regulating chloroplast development (Tokuhisa et al., 1998; Kutik et al., 2004; Liu et al., 2008; Duan et al., 2012). Accumulating evidence suggests that the processes responsible for stabilizing and repairing photosynthetic proteins in response to chilling stress are present in plants to maintain the normal physiological function of chloroplasts. Some mutant plants with defects in the synthesis of some photosynthetic proteins exhibit a variety of symptoms, including variegated or pale leaves and growth suppression under chilling stress (Liu et al., 2010; Kong et al., 2014; Morita et al., 2017; Sun et al., 2017). Here, we summarize the impacts of low temperatures on chloroplasts in four sections, including photosynthesis, redox systems, chloroplast structure and development, and we discuss the response and adaptation of plants to chilling stress.

## Effects of Chilling on the Photosynthesis System in Chloroplasts

The response of chloroplasts to low temperatures greatly depends on the ability of photosynthesis to sense chilling (Pfannschmidt, 2003). It’s reported that both membrane and photoreceptors can perceive the change of temperature. The photosynthetic system complexes are located in the thylakoid membrane. Low temperatures can rigidify the thylakoid membrane and slow enzymatic reactions, thereby affecting the activity of the photosynthetic system. The membrane phase under chilling stress largely depends on the lipid composition. Thus, the lipid composition in the membrane of chloroplasts is very important for maintaining plant photosynthesis under low temperatures (which will be described in detail in the third section). Recent studies have reported that the photoreceptor phyB and phototropins act as thermosensory molecules that perceive fluctuating ambient temperatures (Jung et al., 2016; Legris et al., 2016; Fujii et al., 2017). Phototropin 2, a blue light receptor, was found to direct the position of chloroplasts to optimize photosynthesis in cold-avoidance response (Kodama et al., 2008). The effects of low temperatures on photosynthesis mainly include the occurrence of photoinhibition and a reduction in the activity of some enzymes in the Calvin cycle. Low temperatures have been reported to cause thylakoids to uncouple in chilling-sensitive plants, and the H-ATPase is the site of damage (Peeler and Naylor, 1988; Terashima et al., 1989, 1991a,b). These studies imply that chilling can induce a series of physiological and biochemical events in chloroplasts, and both photoreceptors and membrane can perceive the temperature. It would be interesting to investigate how sensors deliver low-temperature signals to downstream factors.

Photoinhibition is a light-induced reduction in the photosynthetic capacity of chloroplasts in plants. Hodgson et al. (1987) described photoinhibition in plants at low temperatures as chill-induced photoinhibition or chill-temperature photoinhibition. Photoinhibition is an important event that occurs under chilling stress; thus, photoinhibition has been extensively examined by many researchers. First, researchers observed PSII damage following a low temperature treatment (Kaniuga and Michalski, 1978; Kaniuga et al., 1979; Shen et al., 1990). Shen et al. (1990) reported that under cold and dark conditions, electron transport on the oxidizing side of PSII was inactivated, which was ascribed to the degradation of extrinsic proteins and a decrease in the manganese content of PSII. The photodamage and repair occurred simultaneously. Chill-induced photoinhibition is partially determined by the effect of temperature on the recovery process, which involves the synthesis of some chloroplast-encoded proteins, notably, the D1 component at the core of the PSII functional center (Greer et al., 1986). Chilling interferes with the normal replacement rate of D1 in the turnover-repair cycle, which results in more serious photodamage (Allen and Ort, 2001). In tomato, the expression of the coding gene of D1 is regulated by *SlWHY1*, which is significantly induced by chilling (Zhuang et al., 2018). For a long time, scientists believed that PSII was the main component of photoinhibition. However, the photoinhibition of PSI was first reported in 1994 (Terashima et al., 1994). The chilling treatment of cucumber leaves under weak light destroyed F_X_, F_A_, F_B_ and possibly the phylloquinone A_1_ in the iron-sulphur centers of PSI (Sonoike et al., 1995). Several studies have shown that PSI photoinhibition occurs under low temperature and weak light conditions and that the chilling-induced damage to PSI is irreversible, which may explain the irreversible damage to photosynthesis caused by low temperatures (Tjus et al., 1998; Teicher et al., 2000; Kudoh and Sonoike, 2002; Zhang and Scheller, 2004; Zhang et al., 2014). However, the chilling-induced photoinhibition of PSI could be alleviated by moderating the activity of PSII (Huang et al., 2016). Photoinhibition is an important self-protective mechanism in the chloroplast to reduce ROS production under low temperature conditions. The photoinhibition of PSII may minimize damage to PSI by decreasing the electron flow to PSI and maintaining PSI in an oxidized state. The photoinhibition of PSI may also be favorable for preventing overreduction on the electron acceptor side.

The rate of CO_2_ assimilation is also reduced under chilling stress. The reductive activation of two key carbon reduction cycle enzymes, fructose-1,6-bisphosphatase (FBPase) and sedoheptulose-1,7-bisphosphatase (SBPase), has been reported to substantially decrease under light-chilling conditions (Sassenrath et al., 1990; Kingston-Smith et al., 1997; Hutchison et al., 2000). The activity of ribulose-1,5-bisphosphate carboxylase/oxygenase (Rubisco) also declines under low temperature conditions. Chilling may damage the Rubisco protein or affect the redox regulation of the larger Rubisco activase isoform (Byrd et al., 1995; Kingston-Smith et al., 1997; Zhang and Portis, 1999). To acclimate to low temperatures, higher amounts of photosynthetic enzymes, such as the enzymes involved in the photosynthetic carbon reduction cycle and sucrose synthesis, are synthesized to compensate for the reduced activities of other enzymes, including Rubisco, SBPase, stromal FBPase, sucrose phosphate synthase and cytosolic FBPase, under chilling stress (Guy et al., 1992; Holaday et al., 1992; Hurry et al., 1994; Strand et al., 1997, 1999; Savitch et al., 2001). Thus, after plants are exposed to low temperatures for a given period of time, photosynthesis shows strong recovery.

## Effects of Low Temperatures on the Redox State in Chloroplasts

Low temperatures that reduce the activities of the photosynthesis system strike a balance between the light and dark reactions of photosynthesis and cause the production of excess electrons in transport chains. These excess electrons are released via transfer to oxygen to produce ROS, which induce oxidative stress and damage. Therefore, ROS-scavenging mechanisms, such as the release of superoxide dismutase (SOD) and the glutathione-ascorbate cycle, are very important for maintaining normal function in chloroplasts under chilling stress. The levels of ROS and the activities of ROS-scavenging enzymes have been shown to increase in chloroplasts from plants grown under chilling stress conditions (Kaniuga et al., 1979; Choi et al., 2002; Hu et al., 2008). Zhou et al. (2006) reported that a chilling-tolerant cucumber cultivar had higher H_2_O_2_-scavenging activity in chloroplasts than a chilling-sensitive cultivar under low temperature conditions. Manipulation of the antioxidative mechanism in chloroplasts can change the tolerance to chilling stress in transgenic plants. Overexpressing both CuZnSOD and ascorbate peroxidase (APX) in the chloroplasts of transgenic plants enhanced plant tolerance to high light and chilling stress (Kim et al., 2003; Lim et al., 2007). Jing et al. (2006) identified a gene encoding a chloroplast-localized peroxiredoxin Q, *SsPrxQ*, in *Suaeda salsa* L. The overexpression of *SsPrxQ* in *Arabidopsis* enhanced plant tolerance to low temperatures. Decreased chloroplast glutathione reductase (*GR*) gene expression in antisense transgenic plants resulted in chilling sensitivity (Shu et al., 2011; Ding et al., 2012). These studies illustrate that maintaining homeostasis in the redox state in chloroplasts is very important for plants under low temperature conditions. More highly active oxygen-scavenging activities in plants may be due to greater tolerance to chilling stress. An altered redox state in chloroplasts due to various treatments, including methyl jasmonate, melatonin, brassinosteroid and acetylsalicylic acid, can affect plant tolerance to low temperatures (Li et al., 2012; Wu et al., 2015; Zhao et al., 2016; Soliman et al., 2018). In wheat, mechano-stimulation applied during the growth period activated the antioxidant system, maintained the homeostasis of ROS, and improved electron transport and photosynthesis rates in plants exposed to chilling stress during the jointing stage (Li et al., 2015).

Although ROS are typically regarded as toxic to cells and can induce damage to chloroplasts via peroxidation of the membrane, inactivation of enzymes and degradation of proteins, ROS are also considered secondary messengers that regulate diverse functions, such as growth, development, and stress responses, in plants (Foreman et al., 2003; Laloi et al., 2004; Foyer and Noctor, 2005; Pitzschke et al., 2006; Miller et al., 2008; Foyer and Noctor, 2009; van Buer et al., 2016). As signals, ROS can regulate the expression of chloroplast and nuclear genes that repair damage due to chilling (which is described in the fourth section). Currently, ROS signaling pathways have not yet been completely unraveled. Some redox-sensitive transcription factors, such as NPR1 and HSFs, may participate in perceiving these signals (Mou et al., 2003; Fedoroff, 2006). Laloi et al. reported that ROS signals may regulate the expression of antioxidant genes by activating the MAPK cascade (Laloi et al., 2004).

## Effects of Low Temperatures on the Structure of the Chloroplast Membrane

Lipid composition is considered to be closely related to chilling tolerance. Phosphatidylglycerol (PG) is the main component of the membrane, and it is crucial for the photosynthetic process in chloroplasts (Somerville, 1995; Wu et al., 1997; Routaboul et al., 2000). The phase of the membrane is affected by temperature and depends on the extent of the unsaturation of fatty acyl chains. The genetic manipulation of fatty acid (FA) unsaturation has been shown to alter the sensitivity of transgenic plants to chilling (Murata et al., 1992). Levels of unsaturated FAs are regulated by FA-desaturase (FAD) activities (D’Angeli and Altamura, 2016). Researchers have found that the extent of the unsaturation of FAs in PG in the thylakoid membrane can protect the photosynthesis system against chilling-induced photoinhibition (Moon et al., 1995; Liu et al., 2008). Galactolipase, which is responsible for the degradation of membrane lipids, is significantly active in chilling-sensitive plants at low temperatures, and then results in the increase of free FA in chloroplasts (Kaniuga, 2008). Additionally, monogalactosyldiacylglycerols (MGDGs), digalactosyldiacylglycerol (DGDG), sulphoquinovosyldiacylglycerols (SQDGs) and phosphatidylcholine (PC) are major lipids in the thylakoid membrane (Pribil et al., 2014; Li and Yu, 2018). Chilling alters the compositions of galactolipid and carotenoid; in particular, chilling causes a reduction in the levels of MGDGs and SQDGs and an increase in lutein. Furthermore, chilling induces the transition of prolamellar body structures from the compacted “closed” type to the looser “open” type in cucumber and tomato, resulting in decreased membrane fluidity (Novitskaya and Trunova, 2000; Degenkolbe et al., 2012; Skupień et al., 2017). In tobacco, following exposure to 8°C for several days, the relative levels of unsaturated FAs, mainly 18:3n-3 FA, in chloroplast envelopes and thylakoids were significantly elevated, which altered membrane fluidity to accommodate the high-level functioning of the photosynthetic apparatus (Peoples et al., 1978; Sakamoto et al., 2004; Khodakovskaya et al., 2006; Popov et al., 2017). As mentioned above, the presence of high levels of unsaturated FAs in chloroplasts increases the tolerance of plants to chilling (Cronan and Roughan, 1987; Murata et al., 1992). Furthermore, upon exposure to chilling, total FAs are increased, phospholipids are preferentially synthesized and the unsaturation degree of DGDG increases in some plants (Saczynska et al., 1993; Partelli et al., 2011; Scotti-Campos et al., 2014; Gu et al., 2017). Lipids have selective distributions in subcellular organelles, membrane leaflets and membrane domains. The lipid composition has a profound impact on the physical properties of membranes. Once lipid homeostasis is lost, corresponding membrane functions and chloroplast morphology are affected.

The chloroplast ultrastructure is also an important factor that is responsible for the functions of chloroplasts under chilling conditions (Wu and Browse, 1995; Chen and Thelen, 2013). Under cold stress, the size and number of chloroplasts have been shown to rapidly increase in wheat, and the length of the photosynthetic membrane and the number of thylakoids have been shown to increase in grana (Venzhik et al., 2014, 2016). Different degrees of low temperatures lead to different degrees of change in the chloroplast ultrastructure in maize, including changes in the volume densities of granal and intergranal thylakoids, plastoglobules, and peripheral reticulum and dimensions (Kutik et al., 2004; Saropulos and Drennan, 2007; Hola et al., 2008). Generally, in chilling-sensitive plants, the initial manifestations of chilling injury mainly include the swelling of chloroplasts and thylakoids and an increase in plastoglobule numbers (Musser et al., 1984; Zbierzak et al., 2013; Karim et al., 2014). Over time, a series of symptoms emerge, including darkening of the stroma, unstacking of the grana, disintegration of the thylakoid membranes and the chloroplast envelope, vesicle accumulation, and chloroplast disintegration (Musser et al., 1984; Kratsch and Wise, 2000; Zbierzak et al., 2013; Karim et al., 2014). In chilling-resistant plants, starch granules continue to decrease over time and finally disappear (Kratsch and Wise, 2000; Zhuang et al., 2018), and more condensed grana disks are present (Garstka et al., 2007). As time increases, a cold acclimation process is initiated, and a broad range of responses contribute to the adaptation of plants to chilling stress. The above-mentioned symptoms are closely related to the accumulation of ROS, the rigidity of membranes and a profound reprogramming of nuclear and chloroplast-encoded gene expression. However, the underlying mechanism that modulates the presence of these symptoms is largely unclear.

## Effects of Chilling on Chloroplast Development

During chloroplast development, the number, size and composition of plastids change (Mullet, 1988). Chilling is an adverse environmental signal that alters processes, such as pigment synthesis, light-energy absorption, and photosynthetic electron transport, inducing changes in the redox state of photosynthesis components and the accumulation of ROS (Gong et al., 2014; Wu et al., 2016; Morita et al., 2017). Some regulatory factors, including ROS, influence various activities, including multiple chloroplast RNA processing steps, ribosome loading and protein translation, further affecting larger sets of chloroplast transcripts and regulating the expression of chloroplast and nuclear genes (Pfannschmidt, 2003; Kupsch et al., 2012; Exposito-Rodriguez et al., 2017). C-repeat/DREB binding factors (CBFs), which act as act as master transcription factors, are rapidly induced within 15 min during chilling stress and in turn activate the expression of a large number of downstream *cold responsive* (*COR*) genes (Chinnusamy et al., 2007; Zhu, 2016; Shi et al., 2018). CBF3 and *COR* genes are sensitive to the redox state of chloroplasts (Kurepin et al., 2013). Moreover, the expression of some *COR* genes is regulated by CBF-independent transcription factors (Shi et al., 2018), such as RNA-binding domain 1 (RBD1) (Wang S. et al., 2016). These changes in chloroplasts are communicated to the nucleus through retrograde signals, including singlet oxygen (Lee et al., 2007; Kim et al., 2012; Mullineaux et al., 2018) and methylerythritol cyclodiphosphate (MEcPP) (Xiao et al., 2012), in *Arabidopsis thaliana* and H_2_O_2_ in *Nicotiana benthamiana* (Exposito-Rodriguez et al., 2017). Retrograde-signaling molecules further activate a broad range of responses and elicit the expression of stress-responsive nuclear-encoded proteins. There may be multiple retrograde signals from chloroplast to nucleus involving in chilling stress and they may work together in plant.

These regulatory mechanisms facilitate the repair and regeneration of proteins damaged by chilling to maintain the normal physiological functions of chloroplasts. Higher chilling-tolerant plants have stronger repair abilities. In chilling-resistant plants, chloroplast development is retarded (Humbeck et al., 1994). In chilling-sensitive plants, cool temperatures promote cool-temperature-induce-chlorosis (CTIC) symptoms in newly emerging leaves or result in different degrees of an albino phenotype (Song et al., 2014; Wu et al., 2016). Various RNA-binding proteins (RBPs), including pentatricopeptide repeat protein (PPR) and ribonucleoproteins (RNPs), are needed for the response to chilling stress to maintain chloroplast development. The loss of *RBP* genes results in yellow or bleached leaves, and some mutants even exhibit delayed seed germination accompanied by pale primary leaves (Kusumi et al., 2011; Kupsch et al., 2012; Gong et al., 2014; Song et al., 2014; Xu et al., 2014; Wang S. et al., 2016; Wu et al., 2016; Zhang et al., 2016; Cui et al., 2018). Moreover, the transcription levels of genes associated with chlorophyll biosynthesis, photosynthesis and chloroplast development are altered by chilling (Gong et al., 2014; Wu et al., 2016). Some proteins have been reported to influence the development of chloroplasts by disturbing photosynthetic electron transfer, regulating the transcript levels of genes involved in chloroplast transcription/translation and photosynthesis and interfering with RNA processing (Liu et al., 2010; Gu et al., 2014; Wang Y. et al., 2016; Morita et al., 2017). Mutations in these genes result in a deficiency in chlorophyll synthesis that causes chlorotic, white or dead leaves to varying degrees at low temperatures, and some mutations even cause severe growth inhibition, such as the temperature-sensitive chlorophyll-deficient rice mutant *tcd5* (Liu et al., 2010; Gu et al., 2014; Wang Y. et al., 2016; Morita et al., 2017). The decrease in chlorophyll content will further affect the ability of chloroplasts to capture light signals. Furthermore, some metabolites, such as sucrose, trehalose, vitamin E etc., are demonstrated to have a protective function for chloroplasts under chilling conditions. *Arabidopsis* mutants with a vitamin-E-deficiency are sensitive to chilling, due to the defective export of photoassimilate (Stitt and Hurry, 2002; Maeda et al., 2006). Overall, the underlying mechanisms that mediate the levels of *RBP*, *TCD5* and other genes with roles in chloroplast development are not well understood.

## Conclusion and Perspectives

Low temperatures represent an important environmental factor affecting plant growth and development. Nonetheless, under these circumstances, plants need to not only survive but also grow and develop. Chloroplasts are the key organelles in which energy is transformed, and they can sense cold signals. Chloroplast redox imbalance, membrane fluidity, as well as phytochromes are thought to be temperature-sensitive indicators. Multiple sensors maybe simultaneously take part in the process of perceiving low temperature. Low temperature induces chloroplast relocation and the cold-avoidance response in a short time. The phyB photoreceptor and phototropins participate in both temperature and light perception, which may further alter cytosolic Ca^2+^ levels. It would be interesting to study the underlying mechanisms that photoreceptors cause an increase in cytoplasmic calcium and transfer signals in low temperature.

**FIGURE 1:**
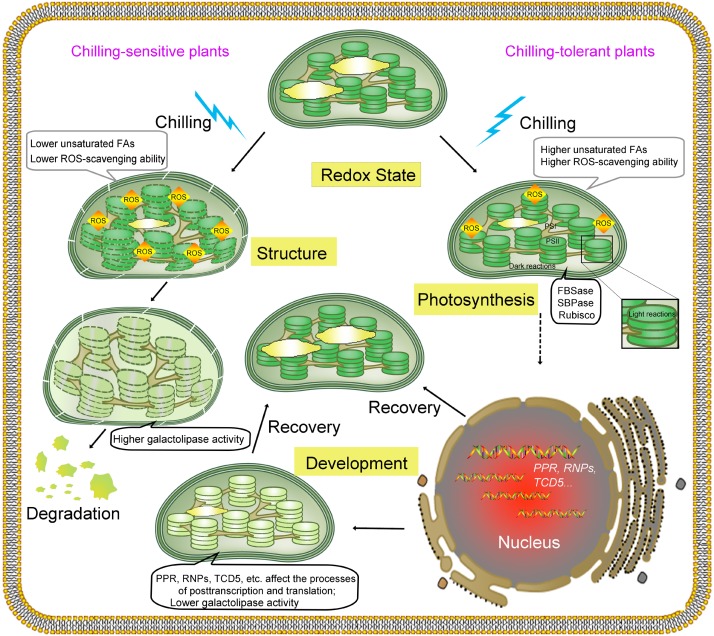
Graphical representation of the effects of chilling on chloroplasts and corresponding responses. Chloroplasts are among the first organelles to sense low temperature, and low temperatures cause an array of changes to chloroplasts. Low temperatures change the membrane state and enzyme activities in chloroplasts, reducing the efficiency of photosynthesis (photoinhibition in PSI and PSII and affecting the Calvin cycle) and leading to the excess production of ROS. On one hand, ROS cause oxidative damage to molecules, including proteins, nucleic acids and lipids. On the other hand, the accumulation of ROS acts as a signal that activates acclimation mechanisms and regulates gene expression in the nucleus and chloroplasts. The activities of two rate-limiting enzymes involved in the Calvin cycle, fructose-1,6-bisphosphatase (FBPase) and sedoheptulose-1,7-bisphosphatase (SBPase), decrease under low temperature. As chilling time increases, the numbers and sizes of the starch grains decrease. In chilling-sensitive plants with lower levels of unsaturated FAs and reduced ROS-scavenging ability, the thylakoid morphology is abnormal, and the membrane structure is disrupted by excess ROS, which is accompanied by significantly higher galactolipase activity; In addition, the morphology of chloroplasts is altered, and the chlorophyll content decreases, which leads to the dysfunction and degradation of chloroplasts. In chilling-tolerant plants, the ROS-scavenging system is activated to protect the membrane against oxidative damage and initiate cold acclimation. Several nuclear genes, such as *PPR*, *RNPs*, and *TCD5*, involved in RNA processing in chloroplasts are regulated at the transcriptional level or the posttranscriptional level, and accompany with lower galactolipase activity. Altogether, these changes help plants better adapt to lower temperatures with or without chlorophyll content change in recovery stage. “Structure” indicates the structure of the chloroplast membrane; “Development” indicates chloroplast development; “Photosynthesis” indicates the photosynthesis system; and “Redox State” indicates the redox state in chloroplasts.

A constitutive low temperature has deleterious effects on plant growth and development. Upon chilling, the activities of enzymes involved in photosynthesis (such as those involved in photoinhibition and the Calvin cycle) and the fluidity of the membrane decrease, affecting the ability of protein complexes to function normally in photosynthesis systems and causing decreased photosynthesis. Once an energy metabolic imbalance occurs, ROS are generated and can accumulate in chloroplasts. ROS, which are oxidative stresses, can destroy the integrity of the membrane (cold-sensitive plants exhibit serious injury and death); additionally, ROS can stimulate the enhancement of ROS-scavenging activities in chloroplasts. Plants must adjust themselves to adapt to ambient temperature. An important step is the retrograde communication between the chloroplast and the nucleus. ROS also act as signals that regulate the transcription and posttranscriptional processing of photosynthesis-related genes in the chloroplast and nucleus to repair or regenerate damaged proteins, maintaining photosynthetic efficiency as much as possible (Pfannschmidt, 2003). The modification of gene transcription, protein translation and transportation to repair chilling-induced damage and induce acclimation to low temperatures leads to changes in chloroplast development, including changes in the size and number of chloroplasts and chlorophyll content. Finally, cold-resistant plants exhibit changes in growth and development to adapt to environmental stress (Figure 1). Chloroplasts play an important role in the process of plant adaptation to low temperature. During this process, plants need to coordinate the relationship between low-temperature adaptation and photosynthetic efficiency in order to obtain better growth and more production. Therefore, there is a trade-off between chilling tolerance and photosynthesis.

## Author Contributions

XL and FB wrote the review. All authors read and approved the final manuscript.

## Conflict of Interest Statement

The authors declare that the research was conducted in the absence of any commercial or financial relationships that could be construed as a potential conflict of interest.
